# Entropy Involved in Fidelity of DNA Replication

**DOI:** 10.1371/journal.pone.0042272

**Published:** 2012-08-09

**Authors:** J. Ricardo Arias-Gonzalez

**Affiliations:** 1 Instituto Madrileño de Estudios Avanzados en Nanociencia, Cantoblanco, Madrid, Spain; 2 Department of Macromolecular Structure, Centro Nacional de Biotecnología, Madrid, Spain; 3 Centro Nacional de Biotecnología–Instituto Madrileño de Estudios Avanzados Nanociencia Associated Unit “Unidad de Nanobiotecnología", Madrid, Spain; Cajal Institute, Consejo Superior de Investigaciones Científicas, Spain

## Abstract

Information has an entropic character which can be analyzed within the framework of the Statistical Theory in molecular systems. R. Landauer and C.H. Bennett showed that a logical copy can be carried out in the limit of no dissipation if the computation is performed sufficiently slowly. Structural and recent single-molecule assays have provided dynamic details of polymerase machinery with insight into information processing. Here, we introduce a rigorous characterization of Shannon Information in biomolecular systems and apply it to DNA replication in the limit of no dissipation. Specifically, we devise an equilibrium pathway in DNA replication to determine the entropy generated in copying the information from a DNA template in the absence of friction. Both the initial state, the free nucleotides randomly distributed in certain concentrations, and the final state, a polymerized strand, are mesoscopic equilibrium states for the nucleotide distribution. We use empirical stacking free energies to calculate the probabilities of incorporation of the nucleotides. The copied strand is, to first order of approximation, a state of independent and non-indentically distributed random variables for which the nucleotide that is incorporated by the polymerase at each step is dictated by the template strand, and to second order of approximation, a state of non-uniformly distributed random variables with nearest-neighbor interactions for which the recognition of secondary structure by the polymerase in the resultant double-stranded polymer determines the entropy of the replicated strand. Two incorporation mechanisms arise naturally and their biological meanings are explained. It is known that replication occurs far from equilibrium and therefore the Shannon entropy here derived represents an upper bound for replication to take place. Likewise, this entropy sets a universal lower bound for the copying fidelity in replication.

## Introduction

Many of the proteins in the cell are molecular motors which move along a molecular track and develop a mechanical work. Most of them work alone and therefore only an individual protein develops a certain task without requiring or optimizing that task by working in cooperation. The single-molecule experimental approach to the study of these motors sheds light on their complex stochastic dynamics and its connection to their biological function [1].

Kinesin is probably the best characterized molecular motor at the single molecule level [2]. It is known that one of the roles of this protein is to transport cargoes along the microtubules with high processivity, that is, to transport a cargo for long distances without detaching from the microtubular track. Polymerases on the other hand have a more complex task. They not only have to translocate along a DNA template but most importantly, they have to copy a DNA single strand so that the fidelity in the so-called polymerization reaction is crucial for the cell division. To do so, DNA/RNA polymerase actually works as both a Turing Machine and a Maxwell's Demon [3]–[6]: it is capable of successively reading one nucleotide at a time, identifying a complementary nucleotide in the environment and writing the information by catalyzing a phosphodiester bond in the nascent replicated strand. Moreover, it is also capable of identifying errors in the copied strand by recognizing the secondary structure of the resulting double-stranded polymer [7]–[9]. Some of these proteins can correct a wrong nucleotide by removing it and resuming the process in that position by the so-called proofreading mechanism [10], and some others include strand displacement activity [11]. DNA polymerase acts as a channel from the information point of view since it passes the genetic information from a template strand to a copied one. The pairing process follows spontaneously by hydrogen bonding and the emerging helical structure of the double-stranded polymer is mainly the result of the stacking interactions between the new base-pair and its immediate previous neighbor in the polymer chain [12].

Kinesin uses the energy from the ATP hydrolysis to move along the microtubules in individual steps of 8 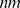 by developing 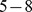 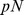 forces, with an efficiency of 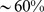 [2]. More complexly, DNA polymerase uses part of the energy from deoxyribonucleotide triphosphates (dATP, dCTP, dGTP and dTTP) hydrolysis for its own motion. Another part of the hydrolysis is used to branch each incorporated nucleotide, that is, it is spent in the phosphodiester bond formation which leads to the nucleobase incorporation in the nascent copied strand. The remaining energy from the triphosphate nucleotides (dNTPs) hydrolysis plus that from the secondary structure formation is still very high what makes paradoxically low the turnover efficiency of this enzyme (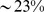) [13]. Besides, it is intriguing that the step of DNA polymerase is much shorter than that of kinesin (0.34 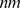) but the forces developed in each step are much higher (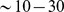 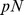 [14], [15]).

Although fidelity is the main role of this enzyme, the energy spent in accurately copying a single strand has only been included in the discussion of the energy balance in the case of independent nucleotide incorporations [16], [17]. Here we calculate the entropy that is needed to order free nucleotides in a reservoir by following a sequence from a DNA template when no dissipation is present. Our theoretical framework allows the natural inclusion of interactions from near neighbors in the replication process. These interactions are closely related to the secondary structure formation of double-stranded DNA and, subsequently, to error recognition by DNA polymerases [7]. On the light of this theoretical framework, we discuss the implication in the energy comsumption by DNA polymerases.

DNA replication is a non-equilibrium process in which dynamical order is naturally generated [18]. Therefore, our calculation marks a lower bound for the energy that must be spent in the ordering process otherwise limiting polymerization. As previously formulated [18], [19], a natural consequence of the present analysis is that DNA polymerase spends an energy in channeling information from a template strand to a copied one with a fidelity which is increased in the presence of dissipation. Our analysis allows envisioning how far from equilibrium this process occurs.

## Analysis

**Figure 1:**
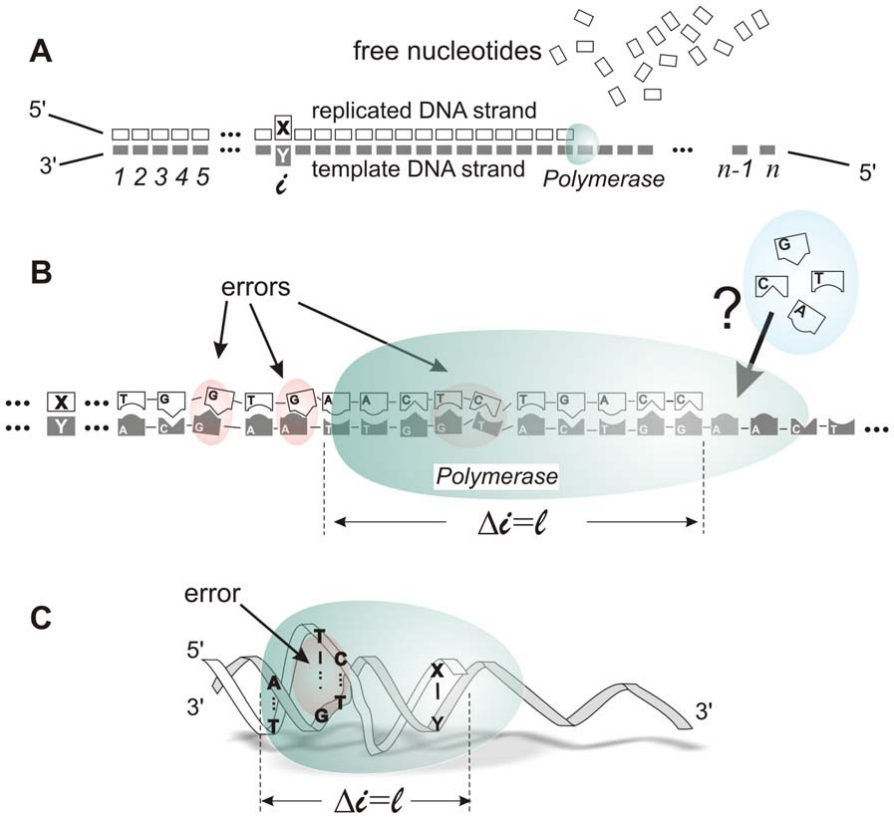
Sketch of DNA replication. (*A*) Variables, 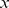, parameters, 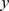, and sequence position 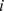 are represented together with 5′ and 3′ ends on both template and replicated strands. (*B*) Polymerase (green) replicating a template DNA strand. Emphasis is placed on the linearity and directionality of the process. 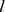 is the number of nucleotides that this nanomachine covers when it is bound to the DNA. (*C*) Polymerase replicating DNA. Emphasis is placed on the recognition of errors based on the sequence-induced secondary structure of the DNA: the structure of DNA polymerase (a ‘palm’, [7]) and dsDNA (a double helix) are evolutionarly adapted to optimally fit each other when Watson-Crick base-pairs are formed. A complete turn of the double helix in B-form is 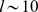 base-pairs.

We start by developing a theoretical framework to analyze information transfer in biomolecular systems. As in former literature [18], [20], [21], we use a mesoscopic approach to study genetic copying at the level of a single DNA polymer. The process of ordering nucleotides according to a prescribed template sequence is shown schematically in Fig. 1*A*. From a thermodynamic point of view, the initial and final states are mesoscopic equilibrium states although, as we study later in this article, the final state is different if the copying process occurs in or out of equilibrium [22]. We will state that a process occurs *in equilibrium* when it takes place through an infinite number of small transitions between equilibrium states. Then, we will say that the polymerase works *in equilibrium* when the nucleotide selection and incorporation procedures performed by this enzyme take place in the absence of friction or other forces which irreversibly release heat [23]–[25]. We suppose that the concentration of dNTPs is larger than that of pyrophosphate (PPi) so that the process we study is phosphate-hydrolysis driven at all time but the motion of the polymerase is very slow so as to preserve equilibrium conditions.

A system which transitions between two equilibrium states can increase its order if the system drives external energy through appropriate dynamical paths [26]. In particular, Andrieux and Gaspard [18] showed that non-equilibrium temporal ordering in copolymerization generates information at the cost of dissipation. Based on experimental evidence from both structural and single-molecule studies, here we mathematically model how DNA polymerase ‘demon’ channels energy from dNTP hydrolysis to order nucleotides according to a template pattern by using a minimum equilibrium description. Our scheme is an idealization of real non-equilibrium copying mechanisms but will lead to a universal (polymerase-independent) entropic upper bound for polymerization to take place.

A sequence in the template strand can be identified as a vector of parameters 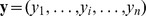, where 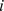 is an index which runs over the nucleotide position and 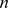 is the number of nucleotides in the template DNA strand. The copied strand is represented here as a sequence of nucleotides given by the vector 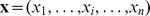 which stems from a multivaluate random variable 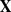. In replication, variables 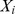 and parameters 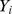 take values over the same alphabet, namely 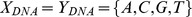. In transcription, they take values over isomorph alphabets, 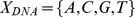 and 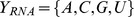. 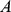, 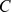, 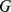, 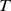 and 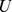 stand for Adenine, Cytosine, Guanine, Thymine and Uracyl nucleotide class, respectively. Hence, we can express without loss of generality for both replication and transcription: 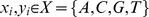.

Since we are only dealing with the ordering process, we do not need to take into account the number of phosphates in the nucleotide or the oxidative state. In the initial state, the nucleotides are independent nucleobase entities with a triphosphate tail and in the final state, they are monophosphate molecular subunits assembled in a linear chain by phosphodiester bonds. However, the nucleobase information remains the same in both cases. In the case of replication, 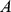, 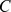, 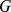 and 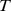 are deoxynucleotides and in transcription 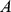, 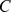, 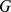 and 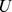 are oxynucleotides. Therefore, as mentioned, we neglect the chemical condition without loss of generality in the *informational* entropy analysis. Exact replication and transcription involve a bijection between variables 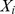 and 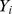 by the so-called Watson-Crick (WC) base-pairing rules, but as we will see, non-WC unions can take place and give rise to copying errors. In translation, the analysis is a bit more difficult since complementarity is replaced by the so-called genetic code which involves a surjective correspondence between variables 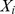 (individual aminoacids) and parameters 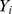 (triads of nucleotides). This case will not be treated here.

The probability of having 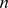 nucleotides in a certain sequence can be expressed as 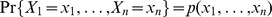. The corresponding entropy is, according to Gibbs formula, 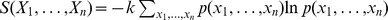, where 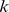 is the Boltzmann constant, “
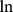
" is the natural logarithm, and the random variables, 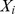, take values, 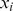, over the genetic alphabet 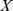. The calculation of these probabilities and their associated entropy involves the very complex analysis of the architecture of genomes and it is similar to that of generating text in a human language. Here, we calculate the entropy of copying the information from a given DNA strand and therefore we are only dealing with the conditional entropy of the sequence 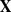 for a given sequence 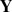. Then, we simply need to use the probabilities of base-pairing nucleotides according to the template sequence, namely 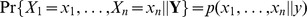, where we have used a double bar to address the conditional character introduced by the complementarity in the base-pair formation. These probabilities can be expressed as a product of conditional probabilities in which each new, incorporated nucleotide, 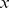, depends not only on the template nucleotide, 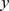, in front but also on the previous base-pairs in the sequence, 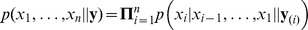, where 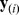 is the parameter vector of the 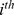 first nucleotides in the template strand. The entropy reads [27]:
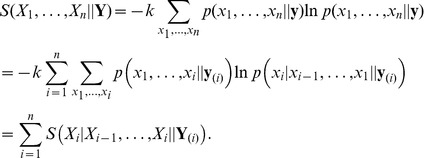
(1)The last part of this equation implies that the total entropy can be expressed as a sum over (double) conditional entropies.

### Polymerase supervision

Polymerization is a spontaneous process at both room and physiological temperatures (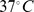) since the free energies of nucleotide incorporation for WC base-pairing are negative. However, the process in the absence of a catalyst may never occur. The biological catalyst or enzyme, the so-called polymerase, is not only able to accelerate the chemical reaction; it also has the capacity for recognizing the secondary structure in the nascent double-helix polymer by a complex mechanism in which the polymerase structure is involved [7]–[9]. Its size covers approximately one helical turn of the double-stranded polymer and this determines a natural length or number of chained nucleotides, 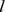, over which correct copying is supervised, as represented in Fig. 1, *B* and *C*. One helical turn involves a number of nucleotides 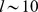. This fact imposes a natural truncation over the conditional probabilities of nearest base-pair neighbors. In other words, polymerase error recognition mechanism can be envisioned as a process in which this molecular machine supervises the copied strand by establishing correlations along 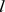 previous base-pairs at each position, 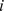, which mathematically involve conditional probabilities. Then, the probability can be approximated by:
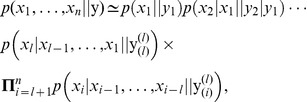
(2)where 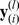 is the parameter vector of 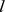 nucleotides which ranges between template positions 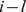 and 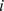. Eq. **1** now establishes:
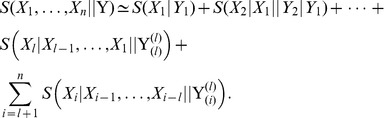
(3)Non-equilibrium paths which increase the fidelity of the copolymerization process are ultimately determined by the above polymerase-DNA structural fitting assumptions. Dynamical time evolutions are therefore concomitant to the basic mechanisms which appear in equilibrium. Then, the equilibrium description will provide an upper entropy bound for DNA replication.

### Entropy and Mutual Information

The total entropy of the final state is 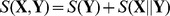. Parameters 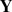 have been fixed throughout evolution and therefore, we assume within the polymerization problem that there is no uncertainty in determining these parameters. Hence, we set 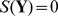 without loss of generality. In these conditions, the final entropy is given by the conditional entropy 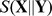, as expressed by Eqs. **1** and **3**.

The mutual information is 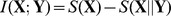 [27], where 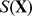 is the entropy of the initial state. 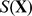 is the entropy of the reservoir and it is fixed for given nucleotide concentrations, as will be addressed later in this article. Then, the lower the entropy of the final state, the higher the information acquired in the copy, the higher the fidelity and the lower the number of copying errors.

Finally, the entropy change in the polymerization process, 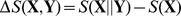, and the mutual information are equal but opposite in sign in these conditions (note that information is not defined in bits), 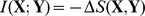.

## Results

The entropy in Eq. **3** does not only depend on the number 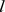 of nucleotides that are imposed by the fitting length of the polymerase to the DNA template but also on the supervising mechanism (e.g. see [9]) that the polymerase establishes by its architecture (molecular structure, e.g. see [7]). Due to the different polymerases that exist in nature and their diverse structure and both polymerization and proofreading mechanisms, not mentioning the cooperative associations of co-factor proteins in eukaryotic replication, the calculation of the entropy cost of copying a nucleotide strand needs of a heuristic model to establish correlations over the 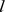 nucleotides that the polymerase supervises. Then, the energy spent in the ordering process involved in polymerization is polymerase-dependent. However, a general upper bound for the entropy can be calculated for all the polymerases based on the fact that the secondary structure of the double-helix of nucleic acids depends majorly on the immediate neighbors [12]. As a first approximation, we calculate the uppest bound by supposing that no influence of the previous base-pairs exist (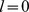). The picture of the polymerase within this approximation is that of a nanomachine which reads one nucleotide at a time and writes a complementary nucleotide to the replicated strand. Later, we introduce the sequence-dependent effects in an either, (1), reversible or, (2), irreversible copying process.

### Entropy of nucleotides ordered with no neighbor influence

The picture of the polymerase within this approximation is shown in Fig. 1*A*. The polymerase only uses the information of one genetic symbol to decide the correct nucleotide to write in the replicated strand, and thus it is represented as if it only covered one position in the template strand. In this case, the random variables 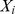 are independent although not identically distributed. Therefore, the probabilites and entropies are:
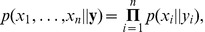
(4)

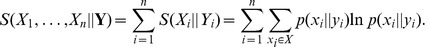
(5)It is important to note that in general the probability, 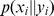, depends implicitly on 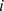 through the random variable 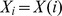 and explicity on the values of the parameters 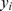. The former dependence implies that the copying process may be subjected to local property changes, such as nucleotide concentration or temperature and ionic gradients, so that the polymerase position 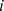 influences the probabilities. The latter dependence is explicit in the values of the parameters 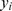 and addresses exclusively the sequence dependence along the template. We assume that polymerization is position-invariant (cf. time-invariant random walk) and then we calculate the entropy from Eq. **5** by using four independent probability distributions, namely 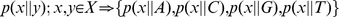. Within this assumption, Eq. **5** becomes
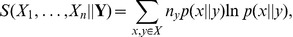
(6)where 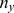 is the number of nucleotides of each type in the template sequence thus fulfilling 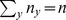. The Shannon entropy of a copied strand is no longer dependent on the template sequence but on the number of nucleotides of each type on the template and its individual hybridization probabilities. The transmitted genetic information would be very poor if replication were taking place in the absence of nearest-neighbor base-pair interactions since a number of sequences 
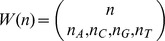
 would be passing the same information. If a dependence on position 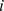 were explicit due to strong local property changes in the environment, the transmitted information would be still poorer since the way boundary conditions affect each replication reaction introduces a further uncertainty. The entropy of nucleotides ordered with no neighbor influence was initially treated by Wolkenshtein and Eliasevich [16] and amended by Davis [17] to introduce wrong incorporations. However these authors did not calculate the error probabilities which we introduce next.

The probability of incorporating a nucleotide 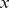 in front of a nucleotide 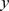 can be estimated from experimental data as
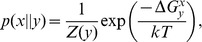
(7)

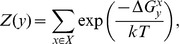
(8)where 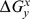 is the energy released (negative) or absorbed (positive) upon pairing a nucleotide 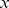 to another 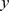 on the template strand and eventual stacking of the newly formed base-pair. Energies 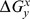 are obtained from experimental data [12] at 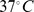 (polymerization occurs *in vivo* in these conditions), as discussed in *Appendix S1*. Then, the probabilities are:
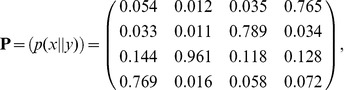
(9)with 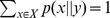. Note that the matrix elements follow the order established by the alphabet sequence 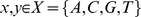. The WC base-pairs appear on the anti-diagonal and, as expected, their propabilities are the largest. Although the real fidelity of a polymerase is much higher than that represented by these probability values, many of its features are addressed by this matrix. Namely, as described in [28] for the human mitochondrial DNA polymerase, misincorporations which involve a 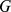 are clearly favored and, with the exception of 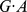, the error 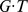 is the most common. With the same exception, incorporations onto 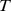 and 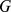 are favored over 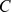 and 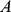. As also measured by [28], the error 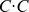 is the least probable. Moreover, it is found a discrimination between misincorporations 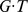 and 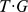. A significant conclusion from this calculation is that errors in polymerization are mainly determined by the thermodynamic affinity of nucleobases.

A similar discussion based on the raw free energy measurements instead of their associated probabilities was formerly established in [29]. The role of kinetic and steric influence in replication fidelity and the importance of mismatch repair in error propagation was also therein discussed in the context of these thermodynamic data. The effect of water exclusion in the active site of DNA polymerases has also been studied. In particular, base-pair interactions were shown to be stronger than would otherwise be expected [30], [31], what should enhance the contrast among the probability values in matrix Eq. **9**. Water exclusion in the DNA double helix has also been shown to decrease the axial base-stacking interactions, as reflected in the DNA stretch modulus [32]. It is therefore expected a type-dependent polymerase mechanism that optimizes fidelity towards reported values of 1 error out of 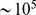 incorporated bases [28]. The presence of exonuclease activity would enhance fidelity towards reported values of 1 error out of 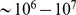 incorporated bases [28]. Both reactions have been reported to be out of equilbrium. Although not reaching these values, the entropy of the polymerized strand in equilibrium is lower than that represented by Eq. **9** (i.e. the transmitted information is higher) due to the presence of nearest-neighbor interactions between base-pairs. We analyze this influence in the next section.

The entropy when no-neighbor interactions are present for a ‘class’ of DNA templates (i.e. with fixed 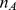, 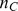, 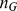 and 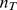) is calculated by introducing the matrix values of Eq. **9** in Eq. **6** and by using the symbol parameters 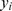, 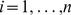. A representative, template-independent value of the absolute entropy of polymerization within this approximation can be calculated in the limit of uniform incorporation of nucleotides. This calculation is performed in *Appendix S2* by using the formalism of *stationary random walk* [27] and the result is 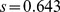 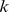 per nucleotide (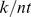).

### Entropy of nucleotides ordered within nearest-neighbor influence

In this section we consider that the incorporation of a new nucleotide depends not only on the nucleotide at position 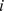 in the template but also on the recently formed base-pair at position 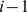. The physical nature of this dependence is the base-stacking interactions between base-pairs which make more probable to place a new nucleotide by a WC union than other combination since the eventual secondary structure of the resulting double-stranded polymer is more stable. The nearest-neighbor interactions implicitly make both the probability and the entropy of the replicated strand become sequence-specific and increase the fidelity of the transmitted information. As pointed out before, the fact that a nearest-neihgbor approximation is sufficient to address secondary structure effects in the hybridization of two strands is supported by former literature [12]. Therefore we introduce the hybridization energies from [12] as coefficients 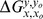, which represent the free energy of positioning a nucleotide 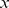 in front of a template nucleotide 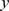 when the previously formed base-pair is 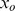 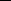 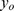, to calculate the Shannon entropy of a replicated DNA strand.

We assume that the polymerase is able to recognize the secondary structure of the double-stranded polymer, as represented in Fig. 1*C*, and thus decide the best match for each incorporated nucleotide. In doing this assumption, we use the fact that a polymerase is continuously grabbing nucleotides at random, fluctuating between an open and close conformation. The polymerase-dNTP binding energy stabilizes a close conformation of the enzyme which is used to attempt the incorporation of each grabbed nucleotide to the template at each position 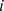. Wrong matches are released in their initial triphosphate state and best matches are hydrolyzed with release of PPi and eventually branched to the previously incorporated nucleotide in the growing complementary strand through a polymerase-catalyzed phosphodiester bond. The fluctuating state of the polymerase is restored after the nucleotide incorporation by using part of the energy from the dNTP hydrolysis. This structural reset enables the enzyme to translocate to the next template position [14], [33] and leads to its memory erasure [6], [24], [25].

Polymerization can be considered a reversible reaction if the polymerase is not included in the process. The inverse reaction, the logical unreading [19] in which a branched nucleotide in its monophosphate state (dMNP) is unbranched and released in a triphosphate state is the so-called pyrophosphorolysis, which is not biologically related to an editing process. The occurrence of this reaction depends on the concentration of PPi in the reservoir, which we suppose to be low compared to that of dNTPs. In the exonucleolysis reaction, which is performed by a different enzyme or by the ‘exo’ domain of some polymerases [10], the initial state of the DNA template is recovered but not that of the cleaved nucleotide since it is released in a monophosphate state with the consequent dissipation of part of the energy from the phosphodiester bond breakage. Exonucleolysis is thus irreversible, as expected from an editing process [24], [25]. However, having in mind that the reservoir is not affected by the substitution of a few dNTPs for dNMPs, the total effect of polymerization and exonucleolysis can be assumed to encompass a reversible copying process. This assumption will become clearer later.

Based on the previous-neighbor-influence assumption, the conditional probabilities are truncated for 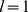 in Eqs. **2** and **3**. Then, the probability distributions are given by coefficients 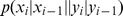, such that 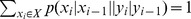, which implies that there is at least one nucleotide 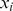 that binds to a nucleotide 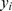 for a previously formed base-pair made up of a nucleotide 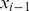 in front of 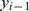. It is also assumed that the energies for nearest neighbors fulfill 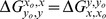 (strand symmetry). This assumption is true provided that hybridization energies do not show a higher-order dependence on the nearest neighbors within experimental resolution [12]. It is approximated otherwise [34].

The total energy of a configuration, 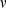, made up of a sequence 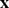 hybridized on a template sequence 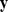 is 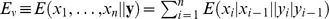 (see *Appendix S3*), where 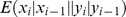 is the energy of pairing nucleotide 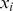 on 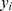 provided that the previously incorporated nucleotide 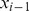 is already hybridized on the template nucleotide 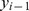. If the energies are not affected by local changes, such as nucleotide concentration or temperature and ionic gradients, their values will only depend on the position, 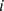, on the template through the values of 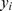.

The Shannon entropy can be calculated by using a partition function formalism, according to a hereafter labeled as *Ising mechanism*, or by using a Markov chain formalism, according to a hereafter labeled as *Turing mechanism*. Figure S1 provides thermodynamic analysis of the entropy.

#### Ising and Turing mechanisms

The Ising mechanism corresponds to an Ising model and it is thus calculated by using a partition function. As we show below, the degree of reversibility of this mechanism depends on the stability of the base-pairs as represented by their free energies. The conditional probability at each step is given by (see *Appendix S3*):
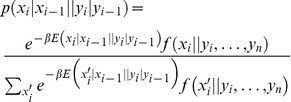
(10)where
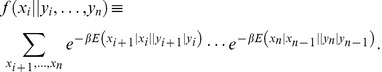
(11)The Turing mechanism, which as mentioned is based on the Markov chain formalism, is implicitly irreversible. The probability of placing a nucleotide 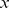 in front of a template nucleotide 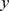 at position 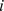 within this formalism is given by (see *Appendix S3*):
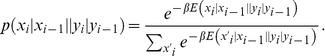
(12)
Eq. **10** represents a probability which depends on the index 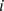 through the symbol value 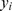 and through the length 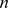 of the template strand chain. Therefore, in the Ising mechanism, for which nucleotides are assumed to freely branch and unbranch, the final macroscopic state is affected by the finite length of the genome. In the Turing mechanism, Eq. **12**, on the contrary, the probability only depends on the position 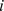 through the sequence. The latter is the process which takes place in polymerization in the absence of exonucleolysis because it is associated to a unidireccional incorporation of nucleotides. The former allows the already incorporated nucleotides to be replaced by new nucleotides and therefore it naturally introduces the effect of exonucleolysis in equilibrium. In the limit of very negative free energies for WC base-pairs (high stability) and very positive free enegies for wrong base-pairs (low stability), the probabilities in Eq. **10** and **12** become Kronecker delta-like functions (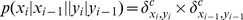, where 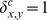 if 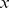 is WC-complementary to 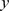 and zero otherwise) and both calculations converge to 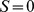, that is, information transmitted in the absence of errors.

**Figure 2:**
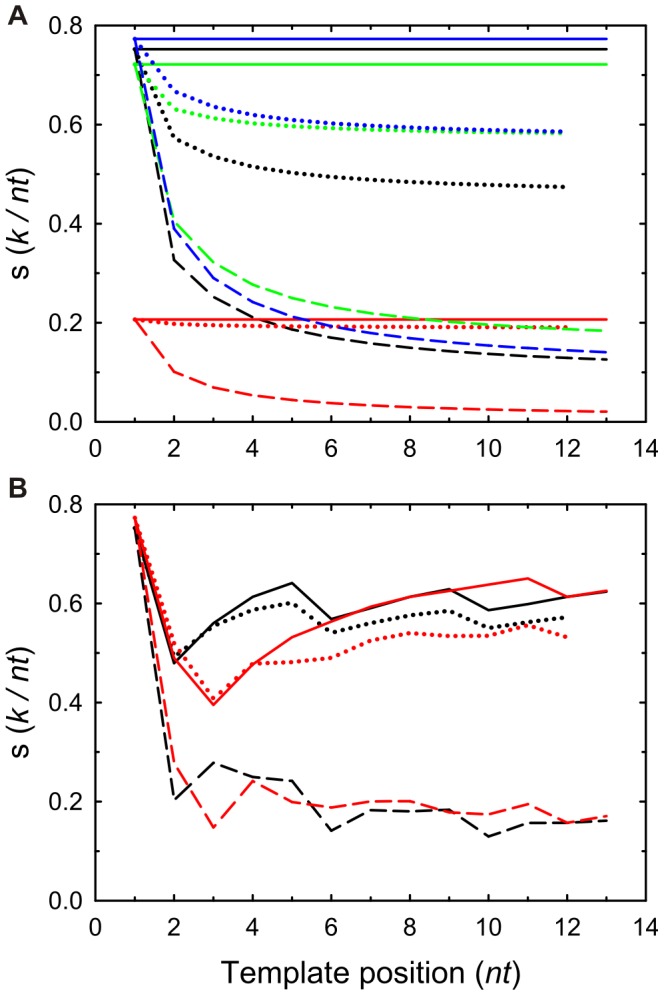
Entropy of replication in equilibrium. Each panel shows the entropy per nucleotide in the absence (solid line) and presence of nearest neighbor influence for an Ising mechanism (dashed lines) and for a Turing mechanism (dotted lines). (*A*) Monotonous template sequences: black lines, polydA; red lines, polydC; green lines, polydG; and blue lines, polydT. (*B*) Black lines, periodic template sequence: ACGTACGTACGTA…; red lines, random template sequence TCCGAGTAGATCT …

**Figure 3:**
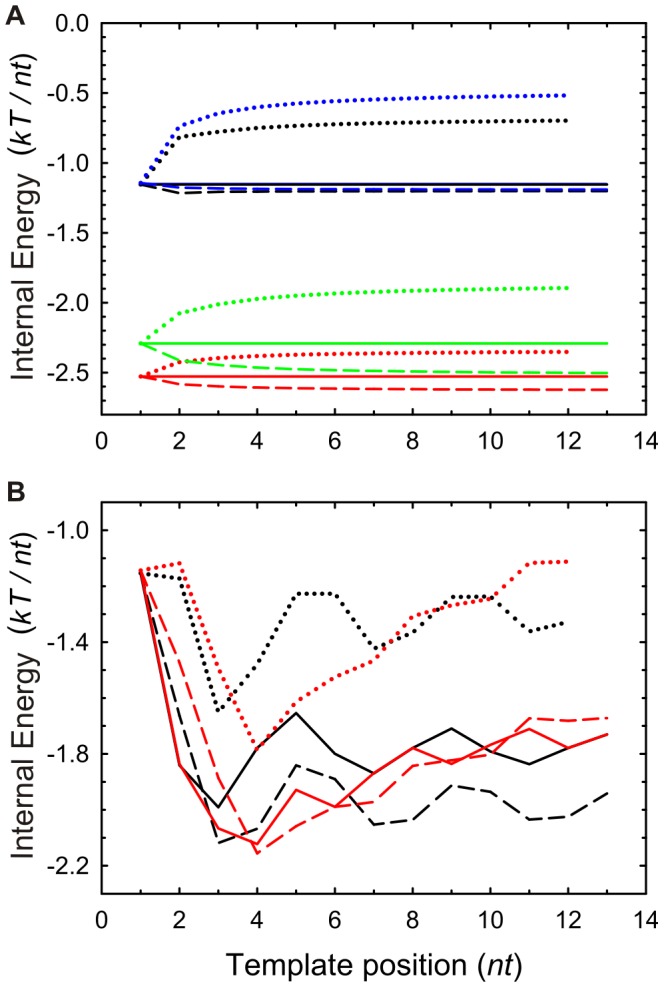
Internal Energy of replication in equilibrium. Each panel shows the mean energy per nucleotide in the absence (solid line) and presence of nearest neighbor influence for an Ising mechanism (dashed lines) and for a Turing mechanism (dotted lines). (*A*) Monotonous template sequences: black lines, polydA; red lines, polydC; green lines, polydG; and blue lines, polydT. (*B*) Black lines, periodic template sequence: ACGTACGTACGTA…; red lines, random template sequence TCCGAGTAGATCT …

**Figure 4:**
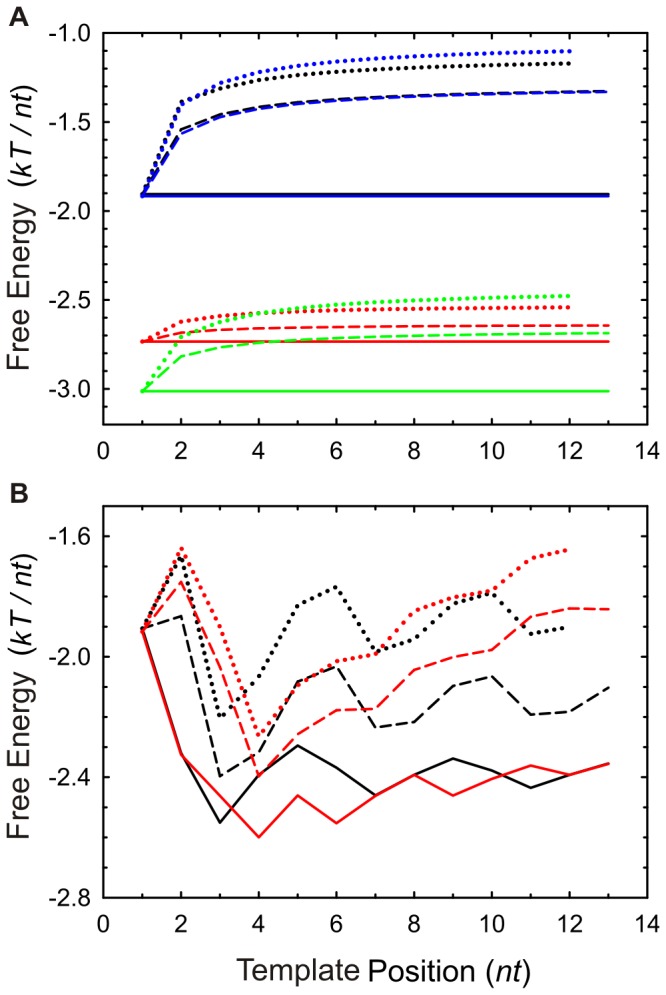
Helmholtz Free Energy of replication in equilibrium. Each panel shows the free energy per nucleotide in the absence (solid line) and presence of nearest neighbor influence for an Ising mechanism (dashed lines) and for a Turing mechanism (dotted lines). (*A*) Monotonous template sequences: black lines, polydA; red lines, polydC; green lines, polydG; and blue lines, polydT. (*B*) Black lines, periodic template sequence: ACGTACGTACGTA…; red lines, random template sequence TCCGAGTAGATCT …

The entropy of replicating monotonous sequences, polydA, polydC, polydG, and polydT, and periodic and random sequences is represented in Fig. 2 for both the Ising and Turing mechanisms. As shown, the absolute entropy per incorporated nucleotide decreases and converges to the thermodynamic limit very rapidly, within 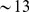 nucleotides, as further confirmed by Montecarlo simulations of the internal energy (see *Appendix S4*). For periodic sequences, the convergence reflects an attenuated periodicity which correlates with the template sequence. This trend is also observed for the internal energy, Fig. 3, and the Helmholtz free energy, Fig. 4. The entropy when no neighbor interaction is assumed is also plotted for comparison. This approximation for independent, non-indentically distributed random variables is not dependent on the kind of calculation and the resulting entropy is always higher than that obtained in the presence of nearest neighbor interactions. This result is expected since when correlations are established among nearest neighbors which lead to conditional probabilities, the probability of error decreases and the absolute entropy is closer to zero [27].

Figure 2 also reflects another feature which takes place when neighbor interactions are taken into account: the absolute entropy for the Ising mechanism is lower than for the Turing one. Although in both mechanisms all configurations are accessible, the number of pathways through which each configuration is accessible in the Turing mechanism is lower. As stated above, the Ising mechanism is reversible and thus it naturally includes the effect of exonucleolysis, what consistently leads to the lowest entropy and consequently to the lowest number of errors in the replicated DNA, in agreement with previous proofreading analysis [19], [35]. Finally, Fig. 2*A* reveals a large entropic discrimination between polymerizing a polydG and a polydC which is not found between a polydA and a polydT. This feature is consistent with what was shown for the case in which no neighbor interactions were taken into account (see matrix Eq. **9** in previous section). This effect is purely entropic since the behavior of the internal and the Helmholtz free energies (Figs. 3 and 4, respectively) do not exhibit such discrimination.

#### Error rates

Errors are defined as non-WC unions. A gross estimation of the probability of error, 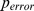, can be obtained through the Shannon-McMillan-Breiman theorem [27], which states that 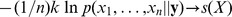 *with probability 1*, where 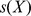 is the entropy per incorporated nucleotide (cf. entropy rate in a random walk). Then, by defining the probability of error from the geometric mean 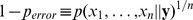, it follows that 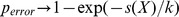. This definition implicitly assumes that each incorporated nucleotide is independent of the previous base-pairs and therefore 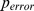 thus obtained represents a higher bound. The entropy per incorporated nucleotide, as extracted from Fig. 2*B* for general random sequences with equal number of nucleotides of each class is 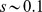 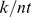 for the reversible process, which leads to 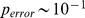.

The probability of error can be more realistically calculated by simulating a large number of sequences according to the joint probability dictated by either the Ising or the Turing mechanism. Montecarlo generation of sequences (see *Appendix S4*) according to the templates studied in Fig. 2 gives rise to 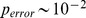 for the Ising mechanism (the lowest average probability of error being for polydC template, 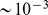) and 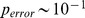 for the Turing mechanism (average probability of error for polydC, 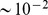). As a cross-check, we note that in the absence of nearest neighbor interactions, these Montecarlo simulations give rise to an average 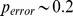 (the lowest 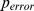, again, for polydC template, 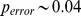), consistent with the information provided by matrix Eq. **9**.

The average probability of error therefore decreases, firstly, in the presence of nearest-neighbor interactions and, secondly, for the Ising mechanism since, as explained above, this mechanism contains the effects of exonucleolysis. Although the average probabilities of error in real, non-equilibrium replication, can be much lower than the ones calculated here in equilibrium, similar error rates have been reported for some polymerases [36].

### Internal and Free Energies

Figure 3 shows the behavior of the mean energy (see *Appendix S3*) which is released upon incorporating a new nucleotide at each step of the polymerase. The stored information in the double-stranded polymer gives rise to a higher internal energy in absolute value for the Ising mechanism than for the Turing one since the number of WC unions, which involve stronger interactions than other pairing possibilities, is higher for the former mechanism.

An error decreases the stability of a microstate (i.e. decreases in absolute value the (negative) energy, 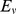, of an individual nucleotide arrangement, 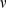, in the copied strand) with respect to the case of a correct (WC) match, not only by contributing with an either less negative or positive energy at the position of incorporation, 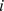, but also at the next step, 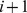, independently of whether the next incorporated nucleotide is a correct match or another error. This does not happen for the case in which no nearest-neighbor influence is taken into account since in that case an error only affects the stability of a microstate at the position where the wrong nucleotide is incorporated. Therefore, if the number of errors when the influence of previous neighbors is taken into account is not much smaller than for the case in which no influence is taken into account, the total energy of a microstate will be on average lower (i.e. will give rise to a less stable configuration) for the former. This is why the internal energy for the Turing mechanism is lower in absolute value compared to that in the absence of nearest-neighbor interactions. The internal energy for the Ising mechanism is however higher in absolute value than for the case in which no nearest-neighbor interactions are taken into account because in this mechanism the number of errors is much smaller than for the Turing one (see Fig. 3).

The Helmholtz free energy, Fig. 4, reflects that the information generated under the Ising mechanism is more significant than that generated under the Turing one since the former produces a smaller number of errors. The free energy also provides information about the spontaneity of copying a template DNA strand. As shown, for a given initial free energy it is more favorable to write a DNA replicate under a process in which each copied symbol does not have a memory of the copying history. When neighboring interactions are considered, the process for which nucleotides are not written by following a directionality (Ising mechanism) is favored over that in which the symbols must be copied on a directional one-after-one basis (Turing mechanism) in the 3′ to 5′ template sense. It is important to note however that the real, non-equilibrium process involves a more complex enzymatic coordination for the former procedure, what actually involves a different physical pathway that could make such procedure become more unfavorable (e.g. see [7], [10], [36]).

### Initial entropy of the nucleotides from the reservoir

The entropy of the initial state is determined by a distribution of probability that a certain nucleotide is grabbed by the polymerase. Hence, this entropy addresses the order in which individual nucleotides reach the ‘pol’ site of the enzyme with independence of whether they would be eventually incorporated to the replicated strand or discarded back to the reservoir. This entropy is not unique and depends majorly on the concentrations of the nucleotides. For a reservoir saturated with each nucleotide class, the entropy can be calculated by the Boltzmann formula, 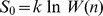, where 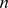 is the number of microstates compatible with a ‘macrostate’ 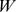. The nucleotide numbers, 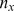, 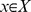, fulfills 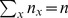 and therefore 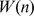 is given by the multinomial coefficient. As expected, for large 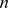, Gibbs and Boltzmann formula provide the same values, namely
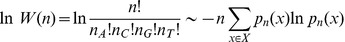
(13)where 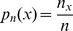 is the probability of grabbing a nucleotide of type 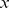 which is in the reservoir at concentration 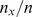. A similar approach to this entropy can be extracted from the case of the ideal gas. The *informational* contribution to the entropy for this system is the same, as expected, and has been analyzed in *Appendix S5*.

By setting equal concentrations for all the nucleotide types, the initial entropy is 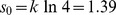 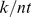. This is the value addressed by setting 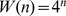, which better describes the infinite number nucleotides of each class contained in an ideal reservoir. The entropy difference between the initial and final states is therefore 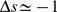 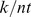. This entropy change cannot be much larger than this value since the lowest (non-equilibrium) final entropy is in any case 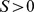. However, approaching 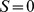, or zero error rate, involves an ever increasing energetic cost with subsequent dissipation [18], [19], in accord with the third law of thermodynamics.

## Discussion

A common mechanical action of linear molecular motors such as kinesin and polymerases is translocation along a molecular track. However, the main role of polymerases is the copying fidelity of the DNA, being this double-stranded polymer a particular molecular track which stores information. The selection of one correct nucleotide at each translocating step of the polymerase constitutes a mechanism which needs of energy as well. We have calculated the entropy balance of a system of nucleotides randomly distributed in a reservoir which are finally incorporated into a copied strand according to a template DNA in the absence of energy dissipation. To that end, we have evaluated the Shannon entropy at both the initial and the final states of the nucleotide symbols by connecting these states through an equilibrium pathway. We show that the entropy related to fidelity must be reduced from the initial state in 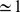 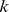 at each step of the polymerase. Given that the initial internal energy of the free nucleotides is 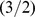 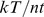 (equipartition theorem), their associated entropy is 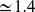 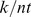, and that the final free energy is that shown in Fig. 4, the free energy invested in copying fidelity must be at least 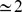 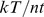.

A gross analysis of the bulk chemical equilibrium between correct/incorrect incorporation of nucleotides shows that the energy required to maintain a copying fidelity of one wrong nucleotide out of 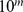, i.e. an error rate 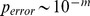, is 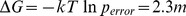 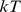 [36], similar to but larger than the above analysis for a low number of errors (i.e. for 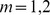 and 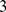) since in this estimation the polymerase is not assumed to work very slowly. In particular, for real error rates of 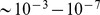, the free energy is much larger than 2 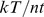. If this energy is added to the thermodynamic efficiency [37] of polymerases, the resulting values would be much higher than those estimated from just the analysis of the translocation mechanism [13]. The contrast between this analysis and the equilibrium polymerization scheme presented in this article demonstrates, on the one hand, that the copying pathway in polymerization (which may be coupled to the translocation one) is far from equilibrium, and on the other hand, that the final state of the nucleotides in the copied strand depends on whether the copying mechanism ocurrs in or out of equilibrium. The latter implies that information managing results in very different fidelities depending on how far from equilibrium the copying mechanism takes place, in agreement with former literature [18], and on which the specific polymerase mechanism is.

Non-equilibrium paths can certainly destroy the information acquired in equilibrium, but they can also amplify it. We have explained that DNA-polymerase structural fitting is responsible for increasing dynamical order in the replication process. Then, the analysis presented here shows a universal higher bound of absolute entropy in polymerization and, subsequently, an error tolerance for the copying fidelity. Each individual polymerase actually uses a specific replication mechanism in the presence or absence of exonucleolysis which sustains an associated error rate evolutionarily coupled to its cellular line development. In our analysis, we include the effects of the previous neighbor base-pair whose physical nature is the base-stacking interactions. These interactions are responsible for the secondary structure of DNA —the double helix— and its correct formation is supervised by the DNA polymerase through structural fitting [7]–[9]. We show that such supervising mechanism reduces the entropy of the copied strand with respect to the case in which these interactions are neglected, a consequence of the fact that information fidelity increases in the presence of conditional probabilities [27].

Finally, we show that the inclusion of the nearest neighbor interactions leads to different absolute entropies of the polymerized strand depending on whether nucleotides are incorporated in either an irreversible or a reversible process. The latter presents the lowest absolute entropy, which is consistent with the error reduction generated by proofreading [19], [35], a mechanism in which nucleotides are removed by exonucleolysis in a backtracking motion of the polymerase or by the presence of an exonuclease enzyme. Error rates within these two equilibrium mechanisms with nearest-neighbor influence are in the 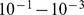, better than the most simple scheme where these interactions are neglected and near some real polymerase fidelities [36]. Most commonly reported polymerases however strongly differ from these rates what ultimately reflects how far from equilibrium they work. The equilibrium mechanisms described here are inherent to more general non-equilibrium polymerization pathways since time evolutions are ultimately mediated by the polymerase demon action.

Although polymerases speed up the replication/transcription reactions, it is important to note that translocation for these molecular motors is slower than for transport molecular motors such as kinesin and myosin [13]. This fact suggests that non-equilibrium replication pathways are mainly focused on the regulation of specific error rates in copying fidelity rather than in the translocation mechanism.

## Conclusions

We have conceived a probabilistic framework based on structural and single-molecule experimental results which models the copy of genetic information by molecular motors through the recognition of DNA secondary structure. The link between thermodynamic entropy, which is based on statistical concepts at the molecular level, and Shannon entropy, which is based on the processing of information, arises naturally within the model. Our mathematical framework provides a connection between entropy and fidelity in replication and leads to universal bounds. Error rates similar to the ones theoretically deduced here in the stepwise equilibrium limit (≲10^−3^) have been measured for some polymerases, what ultimately reflects the consistency of this model with the experiments.

Polymerase ‘pol’ and ‘exo’ catalytic residues are conserved throughout evolution: viral, prokaryotic and eukaryotic polymerases exhibit common structural domains and replication mechanisms. The existence of a certain degree of structural variability and the presence of replicative complexes, which involve auxiliary proteins and coordination strategies, should have an effect on fidelity. In particular, these factors may regulate fidelity to balance maintenance of genetic identity and the species ability to evolve/adapt, in most cases increasing fidelity to several order of magnitude with respect to the values obtained in this work. Our analysis attempts a universal description of polymerase fidelity since only basic assumptions common to all polymerases have been made. This analysis is therefore a starting point for developing theoretical models describing specific polymerases. In this regard, highly processive polymerases which do not require a cooperative association of co-factor proteins like those from some bacteriophages may constitute the first targets for specific modeling.

Developments of the present analysis for specific polymerases can therefore be used to test mechanistic hypothesis on polymerase fidelity by contrasting the subsequently calculated error rates to experimental results. Progresses in this direction are not only interesting to biology but also to inspire nanotechnologies in information processing. Unique to naturally engineered copying/editing nanomachines like the polymerase enzyme is the inherently stochastic mechanisms by which they manage classical information, in contrast to artificial devices for which fluctuations are undesired events. The theoretical modeling of specific polymerases thus represents a physical basis to connect classical information processing in biological systems to artificial, nanoscale platforms, and to open promising avenues in quantum information copying strategies.

## Supporting Information

Figure S1 **Thermodynamic analysis of the Entropy.** (*A*) *Ising Mechanism*: Nucleotides are branched on the template strand without constrainsts of order, direction of replication or number of nucleotides placed at a time. The calculation is based on the partition function formalism. (*B*) *Turing Mechanism*: Nucleotides are branched from the 3′-end to the 5′-end of the template strand on a directional one-after-one basis. The calculation is based on the Markov chain formalism.
(TIF)

Appendix S1 **Hybridization Energies under no neighbor influence.**
(PDF)

Appendix S2 **Entropy per nucleotide for the uniform process.**
(PDF)

Appendix S3 **Partition function vs Markov chain.**
(PDF)

Appendix S4 **Montecarlo simulations.**
(PDF)

Appendix S5 ***Informational*** **and** ***Configurational*** **terms of the Entropy in an ideal gas.**
(PDF)
