# Supplementary figures and images for: Entropy Involved in Fidelity of DNA Replication
Source: PLoS One. 2012 Aug 9;7(8):e42272. doi: 10.1371/journal.pone.0042272 (PMC3415459; doi:10.1371/journal.pone.0042272)

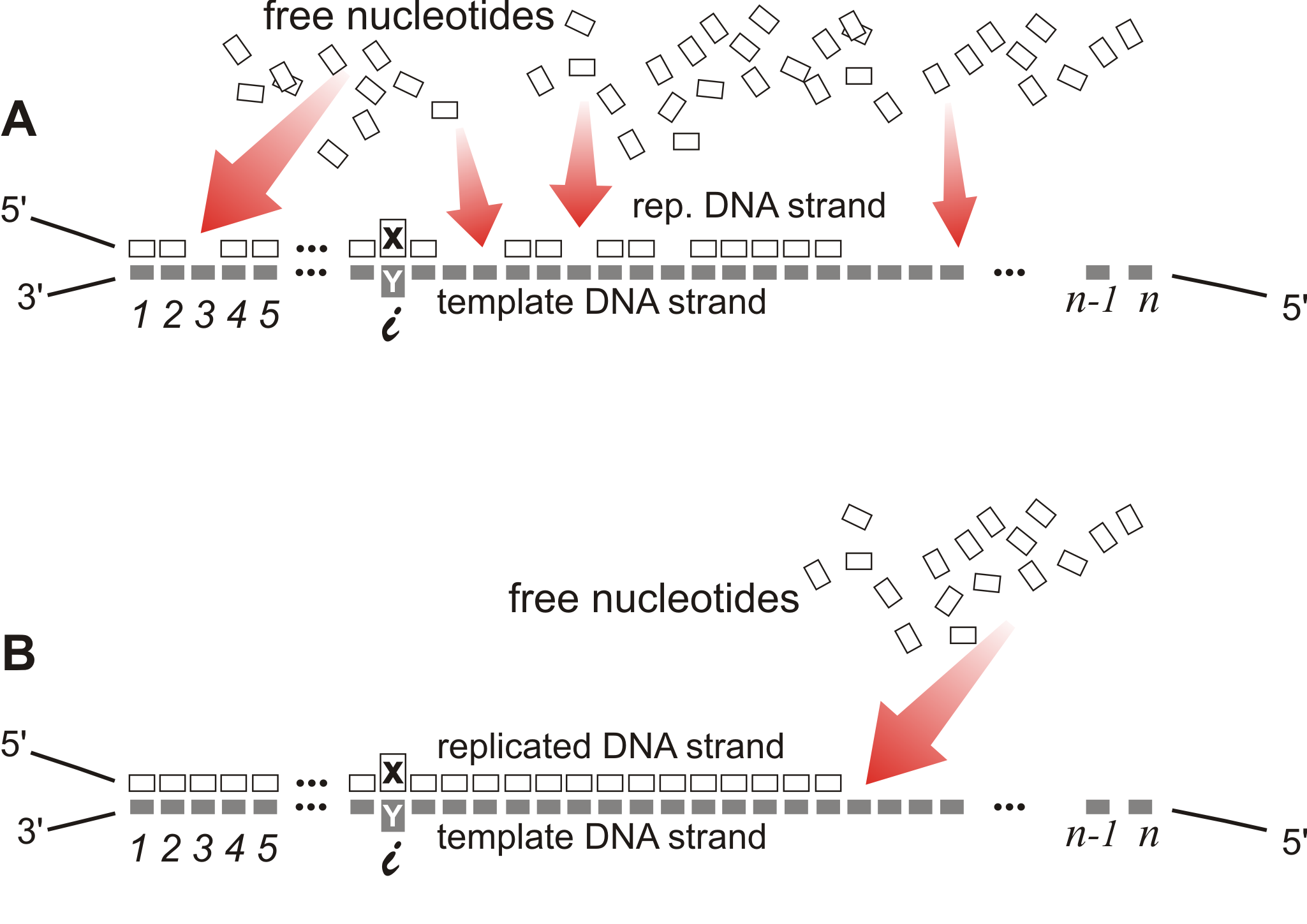

Supplement: Figure S1 — Thermodynamic analysis of the Entropy. (A) Ising Mechanism: Nucleotides are branched on the template strand without constrainsts of order, direction of replication or number of nucleotides placed at a time. The calculation is based on the partition function formalism. (B) Turing Mechanism: Nucleotides are branched from the 3′-end to the 5′-end of the template strand on a directional one-after-one basis. The calculation is based on the Markov chain formalism. (TIF) [file pone.0042272.s001.tif]
