# Supplementary material for: Entropy Involved in Fidelity of DNA Replication
Source: PLoS One. 2012 Aug 9;7(8):e42272. doi: 10.1371/journal.pone.0042272 (PMC3415459; doi:10.1371/journal.pone.0042272)
Supplement: Appendix S1 — Hybridization Energies under no neighbor influence. (PDF) [file pone.0042272.s002.pdf]

Appendix S1

Entropy involved in fidelity of DNA replication

J. Ricardo Arias-Gonzalez[1,2,3,*],

1 Instituto Madrileño de Estudios Avanzados en Nanociencia, Madrid, Spain
2 Centro Nacional de Biotecnología (CNB-CSIC), Madrid, Spain
3 CNB-CSIC-IMDEA Nanociencia Associated Unit "Unidad de Nanobiotecnología"
$\ast$ E-mail: ricardo.arias@imdea.org

Hybridization Energies under no neighbor influence

Free energies ΔG_y^x are obtained from hybridization energies which include the effect of both the base-pairing and base-stacking interactions. The former involve the hydrogen bonding between complementary nucleotides and the latter mainly contain the hydrophobic interaction between the newly formed base-pair and the previous one. These energies have been extensively measured and are summarized in [1] for both correct, Watson-Crick (WC) base-pairs and mismatches. In the approximation that we are dealing with here, we consider that these energy levels are degenerate and assume that the hybridization energy only depends on the unmatched nucleotide y in the template strand. Then, we take averages over all previous base-pair possibilities. For this purpose we define next probability distributions for fixed x-y base-pairs:

$$p_{x,y}(x_0, y_0) = \frac{1}{Z_{x,y}} \exp\left(\frac{-\Delta G_{y_o,y}^{x_o,x}}{kT}\right), \tag{S1.1}$$

$$Z_{x,y} = \sum_{x_o, y_o \in \mathcal{X}} \exp\left(\frac{-\Delta G_{y_o,y}^{x_o,x}}{kT}\right). \tag{S1.2}$$

where $\Delta G_{y_o,y}^{x_o,x}$ is the energy released upon pairing a nucleotide x to another y on the template strand and eventual stacking of the newly formed base-pair with the previous base-pair made up of a nucleotide x_0 in front of another y_0. Most of the base-pairs involving two consecutive non-WC associations are unstable hybridizations and no data were given in [1]. They involve very unfavorable processes (i.e. very high and positive free energies) and we have considered for these cases that $\Delta G = +\infty$. Here we use the data at $37°C$ and 150 mM NaCl concentration since polymerization occurs *in vivo* in these conditions. Then, the hybridization energies in the absence of nearest neighbor interactions are given by

$$\Delta G_y^x = \langle \Delta G_{y_o,y}^{x_o,x} \rangle_{x_0,y_0} = \sum_{x_o, y_o \in \mathcal{X}} p_{x,y}(x_0, y_0) \times \Delta G_{y_o,y}^{x_o,x}. \tag{S1.3}$$

Under these considerations, the free energies in kT units are:

$$\mathbf{\Delta G} = \left(\Delta G_y^x\right) = \begin{pmatrix} 1.01 & 1.70 & 0.34 & -1.65 \\ 1.50 & 1.78 & -2.78 & 1.46 \\ 0.03 & -2.69 & -0.88 & 0.14 \\ -1.64 & 1.39 & -0.16 & 0.71 \end{pmatrix}, \tag{S1.4}$$

where matrix elements follow the order established by the alphabet sequence $x, y \in \mathcal{X} = \{A, C, G, T\}$. Although there is a dependence on both the temperature and the ionic strength, as explained in [1], the latter dependence cancels out in the probability calculation (see Eqs. **7** and **8** in the main text).

References

1. SantaLucia J J, Hicks D (2004) The thermodynamics of DNA structural motifs. Annu Rev Biophys Biomol Struct 33: 415-40.
