# Supplementary material for: Entropy Involved in Fidelity of DNA Replication
Source: PLoS One. 2012 Aug 9;7(8):e42272. doi: 10.1371/journal.pone.0042272 (PMC3415459; doi:10.1371/journal.pone.0042272)
Supplement: Appendix S2 — Entropy per nucleotide for the uniform process. (PDF) [file pone.0042272.s003.pdf]

Appendix S2

Entropy involved in fidelity of DNA replication

J. Ricardo Arias-Gonzalez[1,2,3,*],

1 Instituto Madrileño de Estudios Avanzados en Nanociencia, Madrid, Spain
2 Centro Nacional de Biotecnología (CNB-CSIC), Madrid, Spain
3 CNB-CSIC-IMDEA Nanociencia Associated Unit "Unidad de Nanobiotecnología"
∗ E-mail: ricardo.arias@imdea.org

Entropy per nucleotide for the uniform process

The matrix Eq. **9** in the main text can be interpreted as the probability transition matrix in a four-state Markov process and thus it is possible to calculate the uniform probability distribution. By using this distribution in the limit of uniform incorporation of nucleotides when nearest-neighbor interactions are not taken into account, it is possible to set an upper bound to the absolute entropy per incorporated nucleotide that is generated in the polymerization of a DNA strand starting from a general template DNA. This calculation is useful because this entropy bound does not depend on the DNA template sequence. The uniform distribution fulfills the matrix equation $\mathbf{P}\boldsymbol{\mu} = \boldsymbol{\mu}$ [1] where $\boldsymbol{\mu}$ is the uniform probability (column) vector. In other words:

$$\sum_{y\in\mathcal{X}} p(x||y)\mu(y) = \mu(x) \tag{S2.1}$$

where $p(x||y)$ and $\mu(x)$ are such that $\sum_{x\in\mathcal{X}} p(x||y) = 1$, $\sum_{x\in\mathcal{X}} \mu(x) = 1$. The entropy per incoporated nucleotide (cf. entropy rate in a random walk) for the uniform process, $s(\mathcal{X})$, can be calculated [1] from equation

$$s(\mathcal{X}) = -k \sum_{x,y\in\mathcal{X}} \mu(y)p(x||y)\ln p(x||y). \tag{S2.2}$$

By using the probability transition matrix Eq. **9** we obtain that the absolute entropy per copied nucleotide in DNA polymerization is bounded by $s(\mathcal{X}) = 0.643\ k/nt$ $(37°C)$. This value has been obtained in the limit of, (a), no nearest-neighbor interaction with previously formed base-pairs and, (b), uniform incorporation of nucleotides. This entropic upper bound is independent of the template sequence.

References

1. Cover TM, Thomas JA (1991) Elements of Information Theory. John Wiley & Sons.
