# Supplementary material for: Entropy Involved in Fidelity of DNA Replication
Source: PLoS One. 2012 Aug 9;7(8):e42272. doi: 10.1371/journal.pone.0042272 (PMC3415459; doi:10.1371/journal.pone.0042272)
Supplement: Appendix S3 — Partition function vs Markov chain. (PDF) [file pone.0042272.s004.pdf]

Appendix S3

Entropy involved in fidelity of DNA replication

J. Ricardo Arias-Gonzalez[1,2,3,*],

1 Instituto Madrileño de Estudios Avanzados en Nanociencia, Madrid, Spain
2 Centro Nacional de Biotecnología (CNB-CSIC), Madrid, Spain
3 CNB-CSIC-IMDEA Nanociencia Associated Unit "Unidad de Nanobiotecnología"
∗ E-mail: ricardo.arias@imdea.org

Partition function vs Markov chain

The state, ν, of the system is specified by a sequence of nucleotides $x_1, \ldots, x_n$ replicated in the direction 5' to 3' according to a template composed of an ordered sequence of nucleotides $y_1, \ldots, y_n$ polymerized from the 3'-end to the 5'-end (see Fig. S1), as denoted by:

$$\nu = \{x_1, x_2, \ldots, x_i, \ldots, x_{n-1}, x_n || \mathbf{y}\}, \quad \text{(S3.1)}$$

where $\mathbf{y} \equiv (y_1, y_2, \ldots, y_i, \ldots, y_{n-1}, y_n)$. x_i are variables and y_i parameters such that $x_i, y_i \in \mathcal{X} = \{A, C, G, T\}$. The energy of a state is:

$$E_\nu \equiv E(x_1, \ldots, x_n || \mathbf{y}) = \sum_{i=1}^{n} E\left(x_i | x_{i-1}, \ldots, x_1 || \mathbf{y}_{(i)}\right), \quad \text{(S3.2)}$$

where $\mathbf{y}_{(i)} \equiv (y_i | y_{i-1}, \ldots, y_1)$ and $E(x_i | x_{i-1}, \ldots, x_1 || \mathbf{y}_{(i)})$ is the energy of pairing nucleotide x_i on y_i provided that nucleotides $(x_1, \ldots x_{i-1})$ are already hybridized on nucleotides $(y_1, \ldots, y_{i-1})$, respectively. The probability of a state is:

$$\begin{aligned} P_\nu &\equiv \Pr\{X_1 = x_1, \ldots, X_n = x_n || \mathbf{Y}\} = p(x_1, \ldots, x_n || \mathbf{y}) \\ &= p(x_1 || y_1) p(x_2 | x_1 || y_2 | y_1) \cdots p(x_n | x_{n-1}, \ldots, x_1 || \mathbf{y}), \end{aligned} \quad \text{(S3.3)}$$

where the last part of the equation is the general expansion of the joint probability as a product of conditional probabilities [1]. The mean energy or internal energy of the system is:

$$\langle E \rangle = \sum_{\nu=1}^{N} P_\nu E_\nu = \sum_{x_1, \ldots, x_n} p(x_1, \ldots, x_n || \mathbf{y}) E(x_1, \ldots, x_n || \mathbf{y}), \quad \text{(S3.4)}$$

where N is the number of microstates. The partition function is:

$$\begin{aligned} Z(\beta, n) &\equiv \sum_{\nu=1}^{N} \exp(-\beta E_\nu) = \sum_{x_1, \ldots, x_n} \exp(-\beta E(x_1, \ldots, x_n || \mathbf{y})) \\ &= \sum_{x_1, \ldots, x_n} \exp\left(-\beta \sum_{i=1}^{n} E\left(x_i | x_{i-1}, \ldots, x_1 || \mathbf{y}_{(i)}\right)\right), \end{aligned} \quad \text{(S3.5)}$$

where $\beta = 1/kT$. The probability of a configuration is thus:

$$P_\nu \equiv Z^{-1}(\beta,n)\exp\left(-\beta E_\nu\right)$$
$$= \frac{\exp\left(-\beta\sum_{i=1}^{n} E\left(x_i|x_{i-1},\ldots,x_1||\mathbf{y}_{(i)}\right)\right)}{\sum_{x'_1,\ldots,x'_n}\exp\left(-\beta\sum_{j=1}^{n} E\left(x'_j|x'_{j-1},\ldots,x'_1||\mathbf{y}_{(j)}\right)\right)}$$
$$= \frac{\prod_{i=1}^{n}\exp\left(-\beta E\left(x_i|x_{i-1},\ldots,x_1||\mathbf{y}_{(i)}\right)\right)}{\sum_{x'_1,\ldots,x'_n}\prod_{j=1}^{n}\exp\left(-\beta E\left(x'_j|x'_{j-1},\ldots,x'_1||\mathbf{y}_{(j)}\right)\right)}. \tag{S3.6}$$

It is important to note that the sums in the denominator over $x'_1,\ldots,x'_n$ are nested and therefore they cannot be factorized as independent sums. In other words, Eq. **S3.6** expands as:

$$P_\nu = \frac{e^{-\beta E(x_1||y_1)}e^{-\beta E(x_2|x_1||y_2|y_1)}\cdots e^{-\beta E(x_n|x_{n-1},\ldots,x_1||\mathbf{y})}}{\sum_{x'_1,\ldots,x'_n} e^{-\beta E\left(x'_1||y_1\right)}e^{-\beta E\left(x'_2|x'_1||y_2|y_1\right)}\cdots e^{-\beta E\left(x'_n|x'_{n-1},\ldots,x'_1||\mathbf{y}\right)}}$$
$$= \frac{e^{-\beta E(x_1||y_1)}e^{-\beta E(x_2|x_1||y_2|y_1)}\cdots e^{-\beta E(x_n|x_{n-1},\ldots,x_1||\mathbf{y})}}{\sum_{x'_1}\left[e^{-\beta E\left(x'_1||y_1\right)}\sum_{x'_2}\left[e^{-\beta E\left(x'_2|x'_1||y_2|y_1\right)}\cdots\sum_{x'_n}\left[e^{-\beta E\left(x'_n|x'_{n-1},\cdots,x'_1||\mathbf{y}\right)}\right]_{x'_n}\cdots\right]_{x'_2}\right]_{x'_1}}. \tag{S3.7}$$

The general term of the expansion of $p(x_1,\ldots,x_n||\mathbf{y})$ as a product of conditional probabilities (see Eq. **S3.3**) is:

$$p\left(x_i|x_{i-1},\ldots,x_1||\mathbf{y}_{(i)}\right) = \frac{e^{-\beta E\left(x_i|x_{i-1},\ldots,x_1||\mathbf{y}_{(i)}\right)}f\left(x_1,\ldots,x_{i-1},x_i||\mathbf{y}\right)}{\sum_{x'_i} e^{-\beta E\left(x'_i|x_{i-1},\ldots,x_1||\mathbf{y}_{(i)}\right)}f\left(x_1,\ldots,x_{i-1},x'_i||\mathbf{y}\right)}, \tag{S3.8}$$

where

$$f\left(x_1,\ldots,x_i||\mathbf{y}\right) \equiv \sum_{x_{i+1},\ldots,x_n} e^{-\beta E\left(x_{i+1}|x_i,\ldots,x_1||\mathbf{y}^{(i+1)}\right)}\cdots e^{-\beta E(x_n|x_{n-1},\ldots,x_1||\mathbf{y})}, \tag{S3.9}$$

fulfilling $\sum_{x_i} p(x_i|x_{i-1},\ldots,x_1||\mathbf{y}_{(i)}) = 1$. For the last nucleotide in the chain, $i = n$, it follows that $f(x_n||y_n) = 1$ and

$$p\left(x_n|x_{n-1},\ldots,x_1||\mathbf{y}\right) = \frac{e^{-\beta E(x_n|x_{n-1},\ldots,x_1||\mathbf{y})}}{\sum_{x'_n} e^{-\beta E(x'_n|x_{n-1},\ldots,x_1||\mathbf{y})}}; \tag{S3.10}$$

but, in general, $f(x_1,\ldots,x_i||y_1,\ldots,y_i) \neq 1$ and, therefore, the conditional probabilities depend on the index i, i.e. the position in the polymer chain.

The partition function calculation represents an *Ising mechanism* in which the nucleotides are placed arbitrarily: neither order nor one-by-one basis is implied in this replication procedure (Fig. S1*A*). Unlike this calculation, the Markov Chain calculation implies a *Turing Machine mechanism* in which nucleotides are placed on a one-after-one basis in the 3' to 5' template direction (Fig. S1*B*). This mechanism constraints the sequence in which the different configurations are accessible and therefore, as shown in the main text, the absolute entropy is higher than that from the Ising mechanism. The probability of placing nucleotide x_i onto y_i within this scheme is given by:

$$p\left(x_i|x_{i-1},\ldots,x_1||\mathbf{y}_{(i)}\right) = \frac{e^{-\beta E\left(x_i|x_{i-1},\ldots,x_1||\mathbf{y}_{(i)}\right)}}{\sum_{x'_i} e^{-\beta E\left(x'_i|x_{i-1},\ldots,x_1||\mathbf{y}_{(i)}\right)}}. \tag{S3.11}$$

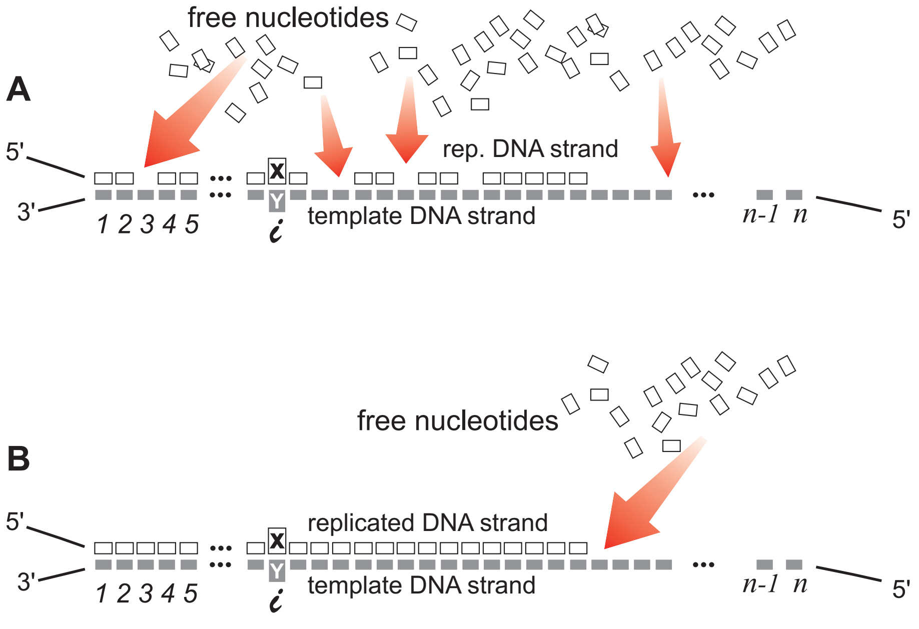

Figure S1. Thermodynamic analysis of the Entropy. (*A*) *Ising Mechanism*: Nucleotides are branched on the template strand without constrainsts of order, direction of replication or number of nucleotides placed at a time. The calculation is based on the partition function formalism. (*B*) *Turing Mechanism*: Nucleotides are branched from the 3'-end to the 5'-end of the template strand on a directional one-after-one basis. The calculation is based on the Markov chain formalism.

Hence, the probability of a configuration ν is given by:

$$P_\nu = p(x_1||y_1)p(x_2|x_1||y_2|y_1)\cdots p(x_n|x_{n-1},\ldots,x_1||\mathbf{y})$$

$$= \left[\frac{e^{-\beta E(x_1||y_1)}}{\sum_{x_1'} e^{-\beta E\left(x_1'||y_1\right)}}\right] \times \left[\frac{e^{-\beta E(x_2|x_1||y_2|y_1)}}{\sum_{x_2'} e^{-\beta E\left(x_2'|x_1||y_2|y_1\right)}}\right] \times \cdots \times \left[\frac{e^{-\beta E(x_n|x_{n-1},\cdots,x_1||\mathbf{y})}}{\sum_{x_n'} e^{-\beta E(x_n'|x_{n-1},\cdots,x_1||\mathbf{y})}}\right] \text{(S3.12)}$$

where we have used brackets to stress that the factorization of the probability here implies that the sums are independent, unlike in the partitition function calculation, Eq. **S3.7**, for which the sum could not be factorized. Note that Eq. **S3.12** also fulfills $\sum_{x_1,\ldots,x_n} p(x_1,\ldots,x_n||\mathbf{y}) = 1$, which is a direct consequence of Eq. **S3.11**.

As explained in the main text, hybridization energies are only dependent on the previously formed base-pair, that is, $l = 1$. If boundary changes, such as nucleotide concentration or temperature and ionic gradients along the DNA polymer, are not present, the energies will only depend on the position, i, on the template through the values of y_i. This means that there is a single set of energies $\Delta G_{y_o,y}^{x_o,x}$, where $x, x_0, y, y_0 \in \mathcal{X} = \{A, C, G, T\}$, and parameters x_0 and y_0 refer to nucleotides preceding x and y, respectively. $\Delta G_{y_o,y}^{x_o,x}$ are the hybridization energy data from [2], also used in previous *Appendix S1*. Note that the fact that the hybridization energies do not depend on i implies that there is also single set of probabilities for the Turing mechanism:

$$p(x|x_0||y|y_0) = \frac{1}{Z(x_0, y, y_0)} \exp\left(\frac{-\Delta G^{x_o,x}_{y_o,y}}{kT}\right), \quad \text{(S3.13)}$$

$$Z(x_0, y, y_0) = \sum_{x \in \mathcal{X}} \exp\left(\frac{-\Delta G^{x_o,x}_{y_o,y}}{kT}\right). \quad \text{(S3.14)}$$

The dependence on the salt cancentration cancels out in this calculation, as was shown for the 0th order approximation (see Eqs. **7** and **8**), so these probability distributions are only temperature-dependent within the empirical expressions given by [2].

For the Ising mechanism, however, the probabilities are affected not only by the sequence parameters y_i but also by the length, n, of the template, and therefore, there is an implicit dependence on i (compare Eqs. **10** and **12** in the main text or Eqs. **S3.8** and **S3.11**).

References

1. Fisz M (1980) Probability Theory and Mathematical Statistics. Krieger Publishing Company.

2. SantaLucia J J, Hicks D (2004) The thermodynamics of DNA structural motifs. Annu Rev Biophys Biomol Struct 33: 415-40.
