# Supplementary material for: Entropy Involved in Fidelity of DNA Replication
Source: PLoS One. 2012 Aug 9;7(8):e42272. doi: 10.1371/journal.pone.0042272 (PMC3415459; doi:10.1371/journal.pone.0042272)
Supplement: Appendix S4 — Montecarlo simulations. (PDF) [file pone.0042272.s005.pdf]

Appendix S4

Entropy involved in fidelity of DNA replication

J. Ricardo Arias-Gonzalez[1,2,3,*],

1 Instituto Madrileño de Estudios Avanzados en Nanociencia, Madrid, Spain
2 Centro Nacional de Biotecnología (CNB-CSIC), Madrid, Spain
3 CNB-CSIC-IMDEA Nanociencia Associated Unit "Unidad de Nanobiotecnología"
∗ E-mail: ricardo.arias@imdea.org

Montecarlo simulations

Simulations of the Internal energy and the probability of error for both the Ising and Turing mechanisms have been generated for template sequences of 20 nucleotides. For the former, the partition function is calculated by using the classical Metropolis-Montecarlo procedure [1]. For the Turing mechanism, each decision step is ruled by the probabilities $p\,(x_i|x_{i-1}||y_i|y_{i-1})$, as established by Eq. **12** in the main text. More in depth, a symbol $x_i \in \mathcal{X} = \{A, C, G, T\}$ and a number $r \in [0,1]$ are chosen at random at step i. Then, considering the outcome of previous step $i-1$ and the template symbol y_i, the symbol x incorporated at position i is that fulfilling the condition $r < p\,(x_i|x_{i-1}||y_i|y_{i-1})$.

For both kind of calculations, averages $\langle E \rangle$ are taken over 10^7 iterations, i.e. over 10^7 sequences generated with each procedure. Comparison of the exact values shown in Fig. 3A with the ones simulated here show a discrepancy within 3.6%, thus confirming a rapid convergence to the thermodynamic limit. The calculations for independent, non-identically distributed variables show discrepancies from the exact calculations in the fifth significant figure for the simulations based on the above procedure, which confirms the validity of the above explained algorithm.

References

1. Chandler D (1987) Introduction to Modern Statistical Mechanics. Oxford University Press.
