# Supplementary material for: Entropy Involved in Fidelity of DNA Replication
Source: PLoS One. 2012 Aug 9;7(8):e42272. doi: 10.1371/journal.pone.0042272 (PMC3415459; doi:10.1371/journal.pone.0042272)
Supplement: Appendix S5 — Informational and Configurational terms of the Entropy in an ideal gas. (PDF) [file pone.0042272.s006.pdf]

Appendix S5

Entropy involved in fidelity of DNA replication

J. Ricardo Arias-Gonzalez[1,2,3,*],

1 Instituto Madrileño de Estudios Avanzados en Nanociencia, Madrid, Spain
2 Centro Nacional de Biotecnología (CNB-CSIC), Madrid, Spain
3 CNB-CSIC-IMDEA Nanociencia Associated Unit "Unidad de Nanobiotecnología"
∗ E-mail: ricardo.arias@imdea.org

Informational and *Configurational* terms of the Entropy in an ideal gas

The entropy per particle of a multicomponent ideal gas can be calculated by using the Sackur-Tetrode formula for a mixture of n particles of types $x \in \mathcal{X} = \{A, C, G, T\}$ and masses m_x occupying a total volume V as follows:

$$\begin{aligned} s &\equiv \frac{S}{n} = \sum_{x\in\mathcal{X}} \left\{ \frac{n_x}{n} k \ln \frac{V}{n_x} + \frac{3}{2}\frac{n_x}{n} k \left[\frac{5}{3} + \ln\left(\frac{2\pi m_x kT}{h^2} \right) \right] \right\} \\ &= -k \sum_{x\in\mathcal{X}} p_n(x) \ln p_n(x) - k \sum_{x\in\mathcal{X}} p_n(x) \ln \left(\frac{nh^3}{V\left(2\pi m_x kT\right)^{3/2}} \right) + \frac{5}{2}k \\ &= s_I(n_x/n) + s_C(x, C) + s_0, \end{aligned} \tag{S5.1}$$

where $p_n(x) = n_x/n$, as defined in the main text. $s_0 = (5/2)k$ is a constant, $s_I(n_x/n) = -k \sum_{x\in\mathcal{X}} p_n(x) \times \ln p_n(x)$ is the herein labeled as *informational entropy* of the initial state and $s_C(x, C)$ is the herein labeled as *configurational entropy*,

$$s_C(x, C) = -k \sum_{x\in\mathcal{X}} p_n(x) \ln\left(C\Lambda_l^3(x)\right), \tag{S5.2}$$

which depends on the type of particle, x, and the total concentration of particles, $C = n/V$. $\Lambda_l(x)$ is a *generalized thermal wavelength* for particles in a liquid, introduced here as:

$$\Lambda_l(x) \equiv \frac{1}{n_l^T(x)} \frac{h}{\sqrt{2\pi m_x kT}}, \tag{S5.3}$$

with $n_l^T(x)$ a *thermal refractive index* of particles, x, in a liquid, which becomes $n_{vac}^T = 1$ for particles in vacuum, since this is the case of the classical ideal gas. In this scheme, the thermal refractive index modifies the thermal wavelength of classical particles in a liquid as $\Lambda_l = \Lambda/n_l^T$, and the dispersion relation as $E = (n_l^T)^{-2} p^2/2m$.

For particles of similar mass, chemical composition and structure, as it is the case of nucleotides, we can assume that both Λ_l and n_l^T are the same for the four types of particles. In these conditions, the configurational entropy is only a function of the total concentration of nucleotides, $s_C(C) = -k \ln\left(C\Lambda_l^3\right)$. The molar concentration of nucleotides in a reservoir for single-molecule experiments and for *in vivo* replication is certainly of $\sim 50\ \mu M$. The mass of the deoxyribonucleotide monophosphates is $m_x \simeq 330\ Da$. Then, the thermal wavelength is $\Lambda \sim 10^{-12}\ m$ and the configurational entropy is $s_C \simeq 26\ k/nt$, which is much larger than the informational entropy, $s_I = 1.39\ k/nt$ (see the main text).
